# Supplementary material for: Patterns of Intron Gain and Loss in Fungi
Source: PLoS Biol. 2004 Nov 30;2(12):e422. doi: 10.1371/journal.pbio.0020422 (PMC532390; doi:10.1371/journal.pbio.0020422)
Supplement: Table S1 — Also available at http://genes.mit.edu/NielsenEtAl/. (4.3 MB ZIP). [file pbio.0020422.st001.zip › NielsenEtAl/html/172.html]

AN5102.1.NCU01164.1.MG07316.1.FG10040.1


```
 CLUSTAL W (1.82) Multiple Sequence Alignments - Introns Inserted


Sequence 1: NCU01164.1	1032 aa
Sequence 2: MG07316.1	1039 aa
Sequence 3: FG10040.1	1034 aa
Sequence 4: AN5102.1	1049 aa
Alignment Length: 1068 aa
Number Identitical Residues: 537 aa
Alignment Score (without introns) 27877


MG07316.1 	-MAEIKIDGKLFQERASHFVNAWKADKRSGDALFGGASSIVVMMGKVEETPEYHKNNAMH
NCU01164.1	-MADIKIDSKVFQERVSHFYNAWKADKRSGDALFGGVSSIVILMGKVDENPEFHKNNAIH
FG10040.1 	-MAEIKIDSKIFQERISHFATAWKNDLRSKDGLFNGAQSLVVMMGKVEEVPEFHKNNAIH
AN5102.1  	MGDEIVIDKTAFFNRLSSFYAAWKADKRSTNSVFGGAGSIIILMGKTDEANSYQKNNAIH
          	   :* ** . * :* * *  *** * ** :.:*.*. *::::***.:*  .::****:*

MG07316.1 	0VSTPQFWLLGYEFPTTLMLFTVDTLYILTTAKKA1KHLDQIKGGRYPVEVLVRGKDAAE
NCU01164.1	0-----FWLLGYEFPTTLMLFTLDTIYILTTQKKA1KYLDQVKGGRYPVEVLVRGKDAAE
FG10040.1 	0-----FWLLGYEFPTTLMLFTLDTLYILTTAKKA1KHLEQLKGGRFPIEVLVRGKDAAE
AN5102.1  	0-----FWLLGYEFPATLFVFTPEVMYVVTTAKKA1KHLEPLKGGKIPVEILVTTKDQEE
          	      *********:**::** :.:*::** *** *:*: :***: *:*:**  **  *

MG07316.1 	NEKAFVKIAEHIKEAG0NKVGVLTKDASKGPFVEEWKKVYTEHCKDVEEVDIAQALSSAA
NCU01164.1	NEKLFIKITDAIKAAG0KKVGVLTKDTSKGPFIDEWKKVYADNCKDVEEVDVAQALSAGA
FG10040.1 	NEKLFVKLTDKIKEAG0NKVGTIAKDTSRGPFVDEWKKVLAEHCKEVSQVDISAALSTYA
AN5102.1  	KTRLFEKCVDIIKSAG0NKVGILPRDTTTGPFVEDWKRVYGKISGDVEEVDISPALSAAC
          	: : * * .: ** ** :*** :.:*:: ***:::**:*  . . :*.:**:: ***: .

MG07316.1 	FSTKDEAEL~RAMRTASKACVALMHPYFLDEMSDILDQEKKVKHSVLAEKVEKKLDDDKF
NCU01164.1	FSVKDETEL~RAMRTSSKACVALLTPYFLDEMSNILDQDKKIKHSALADKVFNKLEDDKF
FG10040.1 	FAVKDESEL~RAMRTASKACVALMTPYFLDEMSNILDAEKKVKHSTLADKVDKKLDDTSF
AN5102.1  	FSVKDTDEL0VSIRNASRACSGLMSDYFVDEMSRLLDEEKQMTHKALSMRIDAKIDDAKF
          	*:.**  **  ::*.:*:** .*:  **:**** :** :*::.*..*: ::  *::* .*

MG07316.1 	WKTVTLPNKQKLPSDFDPEQLDWILGPNVQSGGKYDLRWQTEANNDNLHPGIIVSALGLR
NCU01164.1	WKTVELPNRQKLPADLDPEQLDWILGPIVQSGGKFDLKWQADSDNDILHPGIIIAAMGLR
FG10040.1 	WKTVQLPSKGKLPSDLDPAQLDWILGPAIQSGGKYDLRFAGESNDDNLHAGIIIAAMGLR
AN5102.1  	FN-----KLAKLPSEFDPQQIDWAYGPVIQSGGKYDLKLTAVSDDNNLEPGIIIAGFGIR
          	::     .  ***:::** *:**  ** :*****:**:    :::: *..***::.:*:*

MG07316.1 	YKSYCSSVARTFLVDPNKSQESNYKILHGAHQLILKEVRDGAVVKDVYQKALAYVKSKKP
NCU01164.1	YKSYCSQIARTFMVDPNKSQESNYKFLLAVHNLILKEIRDGAIVKDVYTKAYNFVRSKKP
FG10040.1 	YKSYCSTIARTYLVDPNKAQESSYKLLTLIHNTIIKEIRDGMTAKEVYGRAVGIIKSKKP
AN5102.1  	YKTYSSIIGRTYLVDPTKSQEANYSLLLSVHEAVLKEARDGVVAKELYNKAIGIVRARKP
          	**:*.* :.**::***.*:**:.*.:*   *: ::** ***  .*::* :*   ::::**

MG07316.1 	ELEKHFLKNVGCGIGLEHRDPTMILNAKNTRALKDGMTLCIMTGLQDIENPNPQDKNSKV
NCU01164.1	DLEKHFLKNVGFGIGLENKDPTLILNNKNTRTLKDGMTLVVTTGFSDIQNPNPQDKNSKV
FG10040.1 	EMEKHFLKNVGWGVGLENKDPTLVLNAKNQRVLKDGMTLIINTGFQDIENPHPQDKNSKV
AN5102.1  	ELESHFVKNVGAGIGIELRDSNMILNGKNTRVLKSGMTFSITVGLVDVEEPSVKDKKKNV
          	::*.**:**** *:*:* :*..::** ** *.**.***: : .*: *:::*  :**:.:*

MG07316.1 	YSLVIMDTVRVTASEPVVFTGDAPCDADASSFFFKDEETAQPAPKKEKKESRVGAVATKN
NCU01164.1	YSLILSDTIRVTSSEPVVFTGEAPVDVDATSFFFKDEEEAQPTPKKEKRDSRVGAVATKN
FG10040.1 	YALVLTDTIRVTSSEPVVFTAEAPTSADANSFFFKDDEETEPAPKKEKKDSRVGAVATKN
AN5102.1  	YSMMITDTVRVGEQGPHVFTKDAGIDMDSVSFYFGDEEEPQ-KPAKEKKETKSSAIASRN
          	*:::: **:**  . * *** :*  . *: **:* *:* .:  * ***:::: .*:*::*

MG07316.1 	ITSTRLRSERSTQVD-EDAEKRRREHQKELAAKKQKEGLARFAESTNDQNGTEVKKFKRF
NCU01164.1	ITSTRLRSERNTTVD-EDADKRRREHQKELAQKKQKEGLAKYAESTADENGVEIKKFKRF
FG10040.1 	ITTTRLRSERTTQVANDDIEKKRREHQKELAAKKQREGLARFSESTNDQNGGEVKKFKRF
AN5102.1  	VTRTKLRAERPTQVN-EGAEARRREHQKELAAKKTKEGLDRFAGTTGDDNGVTQKKFKRF
          	:* *:**:** * *  :. : :********* ** :*** ::: :* *:**   ******

MG07316.1 	ESYKRDNQLPPKVKDLAVIVDQKNATVIVPIMGRPVPFHINTIKNASKSDEGEFSFLRIN
NCU01164.1	ESYKRDNQFPPKVKDMGIVIDQKNATIVLPVMGRPVPFHINTIKNASKSDEGEWSFLRIN
FG10040.1 	ESYKRDNQFPVKIKNLEVVVDSKNSTVVLPIMGRPVPFHINTIKNASKSDEGEWSFLRIN
AN5102.1  	ESYKRDNQLPAKVKDLTVYVDHKASTVIVPVMGRPVPFHINTIKNASKSDEGEYAYLRIN
          	********:* *:*:: : :* * :*:::*:**********************:::****

MG07316.1 	FLSPGQGVGRKDDQPFEDATAHFVRSLTFKSLDGDRYTEIANQIANMKKESAKKEQEKKD
NCU01164.1	FLSPGQGVGRKDEQPFEDASAHFVRSLTFKSTDGDRYADIANQISNLKRDAVKKEQEKKD
FG10040.1 	FLSPGQGVGRKDDQPFEDASAHFVRSLTFRSSDGERYNEIATQISNMKRDVVKKEQEKKD
AN5102.1  	FLSPGQGVGRKDDQPFEDLSAHFLRNLTLRSKDNDRFAQVAQDITELRKNALRREQEKKE
          	************:***** :***:*.**::* *.:*: ::* :*::::::  ::*****:

MG07316.1 	MEDVVEQEKLVEIR-~-N1RRPAVLDNVFIRPAMEGKRVPGKVEIHQNGIRYQSPLAAQQ
NCU01164.1	MEDVVEQDKLVEIR-~-N1RRPAVLDNVFIRPAMEGKRVPGKVEIHQNGIRYQSPLSTTQ
FG10040.1 	MEDVVEQDKLVEIR-~-N1RRPAVLDNVYIRPAMEGKRVPGKVEIHQNGIRYISPLNAQH
AN5102.1  	MEDVVEQDKLVEIRK1SD~RRPVKLPDVYLRPPLDGKRVPGEVEIHQNGLRYVSPFRNEH
          	*******:******. :: ***. * :*::**.::******:*******:** **:   :

MG07316.1 	RVDVLFSNVRHLFFQPCQHELIVIIHLHLKDPILIGNKKKTKDIQFYREATDIQFDETGN
NCU01164.1	RVDILFSNVRHLFFQPCQHELIVIIHIHLKDPIIIGNKKKTKDVQFYREATDIQFDETGN
FG10040.1 	RVDVLFSNVKHLFFQPCQHELIVIIHIHLKDPIIVGNKKKTKDVQFYREATDIQFDETGN
AN5102.1  	-VDVLFSNVKHLFFQPCAHELIVLIHVHLKTPIMIG-KRKTRDIQFYREATEMQFDETGN
          	 **:*****:******* *****:**:*** **::* *:**:*:*******::*******

MG07316.1 	RKRKYRYGDEDEFEAEQEERRRRAELDRLFKSFAEKIADAGKSENLEVDMPLRELGFNGV
NCU01164.1	RKRKYRYGDEDEFEAEQEERRRRAELDRLFKSFAEKIAEAGRNEGIEVDMPLRDLGFNGV
FG10040.1 	RKRKYRYGDEDEFEAEQEERRRRAELDRLFQGFAQKIAEAGRNEGIEVDMPIRELGFHGV
AN5102.1  	RRRKHRYGDEEEFEAEQEERRRRAALDREFKAFAEKIADAGKDEGVDVDIPFREIGFTGV
          	*:**:*****:************* *** *:.**:***:**:.*.::**:*:*::** **

MG07316.1 	PFRSNVYIQPTTECLIQITEPPFLVVTLDDIEIAHLERVQ~FGLKNFDLVFVFKDFTRPP
NCU01164.1	PFRSNVYIQPTTECLIQITEPPFMVITLEDIEVAHLERVQ~FGLKNFDLVFVFKDFTRPP
FG10040.1 	PFRSNVFVQPTTDCLIQVVEPPFMVITIEEVEIAHLERVQ~FGLKNFDMVFVFKDFTRAP
AN5102.1  	PNRSNVLIQPTTDALVQLTEPPFLVISLNEIEIAHLERVQ0FGLKNFDLVFVFKDFHRAP
          	* **** :****:.*:*:.****:*::::::*:******* *******:******* *.*

MG07316.1 	VHINTIPVESLEDVKEYLNQSDIAYSEGPLNLNWPTIMKTVTADTHEFFEGGGWSFLQAD
NCU01164.1	YHINTIPVESLEDVKEFLDSSDIAFSEGPLNLNWGVIMKTVTANTHQFFLDGGWGFLQND
FG10040.1 	YHVNTIPVEFLDQVKDYLDSSDIAYTEGPLNLNWPTIMKTVTADTHQFFADGGWSFLQAD
AN5102.1  	VHINTIPVENLEGVKDWLDSVDIAYTEGPLNLNWTTIMKTVVSDPYGFFADGGWSFLAAE
          	 *:****** *: **::*:. ***::******** .*****.::.: ** .***.**  :

MG07316.1 	SDDEGDGEEEEESAFEVSESELEEASESSEEDSEFDSNASDEASDED-ASEEEEGEDWDE
NCU01164.1	SDDDEVEEEEEESAFEIDESELEDASESSEEDSEYDSNASEEASDEAEDSEEEEGEDWDE
FG10040.1 	SDDDGGDPSDEESAFEMDEDEFDEESESSDEGSDFGSNASDDEGSDAELDSEDEGEDWDE
AN5102.1  	SDSEDGSDEEEESAFELSESELAAD-ESSEEDSDYDDDAS--ADDDFSADEDESGEDWDE
          	**.:    .:******:.*.*:    ***:*.*::..:**   ..: . ..::.******

MG07316.1 	LERKAKRKDRESALDDEEEERGNSKKK~QRKR------------------------
NCU01164.1	LERKAKKRDRESGFDDEEE-RAAPKKR~RK--------------------------
FG10040.1 	LERKAKKRDRESAME--EEDRGANKKK~QRKR------------------------
AN5102.1  	LEHQAKKKDRESGLD--DEDRGKKRKS2VNGIFPLQIISDYRNLQKVDMLTYIIWL
          	**::**::****.::  :*.*.  :*   .   . .  :.  . .. .  :
```
